# Large Bottleneck Size in *Cauliflower Mosaic Virus* Populations during Host Plant Colonization

**DOI:** 10.1371/journal.ppat.1000174

**Published:** 2008-10-10

**Authors:** Baptiste Monsion, Rémy Froissart, Yannis Michalakis, Stéphane Blanc

**Affiliations:** 1 UMR BGPI, INRA-CIRAD-SupAgroM, Campus International de Baillarguet, Montpellier, France; 2 GEMI, UMR CNRS–IRD 2724, IRD, Agropolis, Montpellier, France; University of North Carolina, United States of America

## Abstract

The effective size of populations (*Ne*) determines whether selection or genetic drift is the predominant force shaping their genetic structure and evolution. Despite their high mutation rate and rapid evolution, this parameter is poorly documented experimentally in viruses, particularly plant viruses. All available studies, however, have demonstrated the existence of huge within-host demographic fluctuations, drastically reducing *Ne* upon systemic invasion of different organs and tissues. Notably, extreme bottlenecks have been detected at the stage of systemic leaf colonization in all plant viral species investigated so far, sustaining the general idea that some unknown obstacle(s) imposes a barrier on the development of all plant viruses. This idea has important implications, as it appoints genetic drift as a constant major force in plant virus evolution. By co-inoculating several genetic variants of *Cauliflower mosaic virus* into a large number of replicate host plants, and by monitoring their relative frequency within the viral population over the course of the host systemic infection, only minute stochastic variations were detected. This allowed the estimation of the CaMV *Ne* during colonization of successive leaves at several hundreds of viral genomes, a value about 100-fold higher than that reported for any other plant virus investigated so far, and indicated the very limited role played by genetic drift during plant systemic infection by this virus. These results suggest that the barriers that generate bottlenecks in some plant virus species might well not exist, or can be surmounted by other viruses, implying that severe bottlenecks during host colonization do not necessarily apply to all plant-infecting viruses.

## Author Summary

Whether selection or stochastic genetic drift is the major force driving the evolution of a virus depends largely on the size of the viral population, with the former being predominant in large populations and the latter taking over when population sizes are transiently or durably reduced. This question has been intensively debated in both plant and animal viruses, as demographic fluctuations throughout viral life cycles are poorly understood. In plant viruses, an extremely small population size—down to a few founder genome units colonizing each leaf—has been formally estimated in two instances, and all other virus species investigated so far have consistently been shown to undergo extreme demographic bottlenecks during systemic invasion of their host. This situation conveys the general idea that all viruses are confronted with “universal barriers” in plants, imposing repeated transient decreases in their population size, thus making genetic drift a major constant driver of their evolution. Here, using the example of *Cauliflower mosaic virus*, we mitigate this general idea by showing that at least one virus species can overcome such putative limiting barriers and massively invade leaves with hundreds to thousands of founding genome units.

## Introduction

The main forces driving changes of the frequencies of alleles within populations are selection (deterministic) and genetic drift (stochastic). While both forces obviously act concomitantly in most cases, the balance of their respective action is governed by a number of factors, one of the most important being the effective population size (*Ne*) [1]–[3]. This parameter, commonly defined as the size of an ideal population which would drift at the same rate as the observed population [4], is widely investigated in the literature both theoretically [5]–[8] and experimentally [9]–[11] in a broad variety of organisms. *Ne* significantly informs whether the evolution of a given population might be better described by a deterministic or a stochastic evolution model [12]–[14]. In brief, when *Ne* is large, competition between genetic variants is fully acting, with no or little interference of random processes, and selection shapes the genetic composition of corresponding populations. Conversely, in populations with a small *Ne*, various processes resulting in stochastic “sampling” of genetic variants that will engender the next generation are prevalent and counter the effect of selection.

Experimental evolution using viruses as biological models has developed enormously during the past two decades. A series of converging reports on viruses of bacteria, animals, and plants confirmed that protocols maintaining constant large population sizes lead to selection of the fittest variants, thus augmenting the mean fitness of viral populations [15]–[17]. On the other hand, the repeated experimental imposition of severe bottlenecks (temporarily reducing *Ne*) is associated with relaxed selection and strong drift, dramatically reducing the mean fitness in the population [18]–[21]. Viruses are intuitively perceived as having extremely large population sizes during host invasion. While this is true for the *census* population size, i.e. the number of viral genomes accumulating in single hosts, it does not indicate what fraction thereof is actually actively replicating and propagating, or is efficiently progressing into new organs and tissues, yielding subsequent generations upon host colonization. Estimating viral *Ne* during systemic infection of a host has proved experimentally difficult, as illustrated by the impressive number of studies published, and still debated, on HIV [22]. Consequently, virus species for which *Ne* has been formally evaluated during invasion of various organs and tissues of the host remain extremely few in both plants and animals. The question of whether deterministic or stochastic models better explain the evolution of viral populations is thus pending further investigation of *Ne*.

In plants, several specific barriers could impose severe bottlenecks on populations of invading viruses. The level at which the existence of such bottlenecks has been most documented is the colonization of new leaves, not only because of the obvious practical ease of comparing the genetic content of different leaves from the same host, but also because this level results from long-distance movement of the virus population, which is loaded into the vascular system from source leaves and unloaded into sink leaves (for review, see [23]). As discussed later, other phenomena could also generate population bottlenecks, but virus movement within the host plant through plasmodesmata is most often considered as a major putative obstacle to exponential expansion.

While not directly estimating *Ne*, two experimental evolution studies demonstrated the existence of viral population bottlenecks during leaf colonization. With a mixture of 12 engineered genetic variants of *Cucumber mosaic virus* (CMV) co-inoculated into tobacco plants, Li and Roossinck [24] showed that diversity decreased randomly but continuously in the viral population, as increasing numbers of variants were lost when systemic infection progressed into upper, newly-formed leaves. Even more illustrative was the spatial distribution of the genetic diversity of a *Plum pox virus* (PPV) population, maintained in a perennial host tree for over 13 years [25]. While a large number of related PPV variants could be distinguished and detected in various organs and tissues, distinct subpopulations were shown to be isolated in different branches. Extreme population bottlenecks were further evidenced when the virus progressed into newly formed leaves, which were all colonized by one single viral genotype.

The effective size of virus populations during systemic infection of host plants has been evaluated more formally in two instances. Populations of *Wheat streak mosaic virus* (WSMV) invading a wheat tiller [26], and of *Tobacco mosaic virus* (TMV) invading a tobacco leaf [27], were shown to be founded by as few as 4 and 2–20 virus particles, respectively, hence again resulting in severe bottlenecks.

All the studies cited above converged to the conclusion that the population size of plant viruses fluctuates dramatically and can be temporarily remarkably small, i.e. of the order of one to a few genome units founding the population that subsequently develops to billions of genomes in each systemically infected leaf. Such a demographic regime suggests that genetic drift is a major force in plant virus evolution, as proposed and discussed by several authors [24]–[28]. The fact that extreme population bottlenecks are consistently described for unrelated virus species infecting mono- or di-cotyledonous hosts appealingly suggests that all plant viruses might be subject to the same phenomenon [24], [27]–[29], perhaps related to the unavoidable physical barriers that hamper the systemic movement of all viruses in plants. However, this tempting generalisation definitely requires closer inspection as, if proven true, it would illustrate the impossibility of viral adjustments and trade-offs on important traits requiring large *Ne*. In contrast, a single counter example would demonstrate that, in some virus-plant associations, such barriers might not exist or might be surmounted by the virus. Further research on more diverse virus species, with different replication strategies and different life cycles, is still needed in order to assume extremely small *Ne* as a general rule during host plant colonization.

In this report, we assessed the importance of bottlenecks during systemic host colonization in populations of *Cauliflower mosaic virus* (CaMV), a DNA virus whose biological properties differ largely from those of the RNA viruses investigated in the studies cited above. Monitoring the frequency of several engineered allelic variants within leaves of single host plants revealed remarkably small stochastic fluctuations in the genetic structure of numerous CaMV populations, even over considerable periods of time. Beyond demonstrating that CaMV populations are not subject to intense genetic drift, and hence do not undergo severe demographic bottlenecks, the slight stochastic fluctuations detected were exploited to infer the effective size of CaMV populations upon systemic leaf colonization. Our estimates consistently indicate that several hundreds of genome units founded the viral populations in all leaves analyzed (originating from different plants). This value, which is ∼100-fold higher than values previously reported for other plant viruses, demonstrates that extremely small viral *Ne* during host plant colonization is not a general rule and opens up the possibility of trade-offs on viral traits that directly or indirectly depend on *Ne*.

## Materials and Methods

### Engineered CaMV variants

The six plasmids (pCa-VIT1 to pCa-VIT6) used in this study to generate the six CaMV allelic variants have been described in detail and characterized previously [30].

All are infectious full-length clones of the CaMV Cabb-S isolate [31], where a specific genetic marker (a dsDNA non-coding sequence of 40 bp) has been inserted between CaMV ORFs II and III. These markers should not affect any viral function, as non-coding sequences between CaMV ORF do not affect translation. When inoculated individually into turnip plants, each CaMV clone (CaMV-VIT1 to CaMV-VIT6) induces symptoms similar to CaMV wild type, and all six genetic markers have been shown to be stably maintained in the viral genome even after three successive passages in plants [30].

### Plant growth conditions and inoculation with mixed CaMV-VIT populations

All turnip plants (*Brassica rapa* var. “Just Right”) were maintained in an insect-proof greenhouse under controlled conditions: 25/19°C day/night with a photoperiod of 16/8 h day/night.

Plants infected for 21 days with one of the CaMV-VIT1 to -VIT6 clones (one plant per clone) were used to prepare virus particle-enriched fractions as previously described [30]. Equal volumes of each of the six virus particle-enriched fractions were pooled to produce a mixed inoculum (designated Mix6VIT) containing all six allelic variants of CaMV. Fifty healthy young plantlets, at the three-leaf stage, were mechanically infected by rubbing 20 µl of Mix6VIT solution onto the entire surface of the two larger leaves, previously powdered with abrasive carborundum. First symptoms indicative of CaMV infection appeared on non-inoculated leaves within 7 to 9 days, and all plants proved systemically infected at 11 days post-infection (dpi).

### Harvesting of leaf samples

At 13 dpi, i.e. very soon after systemic infection had developed, the inoculated leaves were discarded and all large expanded leaves were collected. In all cases, two to three young expanding or emerging leaves in the centre of the rosette were left unsevered in order to allow continuous growth of the plants for further sampling as described below. The viral population extracted from each plant at this early stage was considered as the initial population.

Thirty-two days later (at 45 dpi) the same plants had grown continuously and produced 10 to 15 newly expanded leaves. At this stage, one single leaf from each plant, randomly chosen between the 5^th^ and the 12^th^ newly formed leaves, was collected. The viral population extracted from each single leaf at this late stage was considered as the final population. All leaf samples were stored at −20°C until further viral DNA purification.

The rationale of this sampling protocol is further discussed below.

### Purification of viral DNA and genetic composition analysis

Purification of viral DNA from the harvested leaf samples, as well as the analysis of the genetic structure of the corresponding viral genome populations were performed exactly as described previously, using the QSS (Quantitative Single-letter Sequencing) method [30]. Briefly, QSS allows the simultaneous quantification of numerous allelic variants in a single DNA sample. After a PCR amplification step using a pair of primers flanking the marker-containing region, the PCR product is submitted to single-letter sequencing primed with a fluorescently-labelled oligonucleotide, located upstream of the markers' position. The resulting monochromatic electropherogram exhibits numerous specific diagnostic peaks, attributable to specific variants, signifying their presence/absence in the DNA sample. Finally, peak fluorescence can be quantified and used to estimate the frequency of the corresponding variant in the DNA population. The accuracy and reproducibility of the QSS method have been fully evaluated and shown to be equivalent or higher than that of competing technologies (including those based on real-time PCR) for quantifying variants with a relative frequency above 5% in the DNA population [30].

### Statistical analysis

Changes in the relative frequencies of the six alleles (-VIT1 to -VIT6) between the initial and final populations, sampled as described above, were precisely monitored in 50 replicate test plants.

In order to calculate the number of founder genomes initiating each final population in a single leaf (N), all parameters accounting for the transition from p (initial frequency of a given marker) to p′ (final frequency of the same marker) must be evaluated. For any given plant the difference between p and p′ (Δp) may be potentially attributed to genetic drift and to selection:
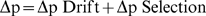
(1)


There is no a priori reason to assume that any selection affecting the frequency of the markers is heterogeneous across plants (e.g. favouring a marker in a plant and selecting against it in another plant). Moreover, given the way our markers were constructed, there is no *a priori* reason to expect directional selection in favour of any of them. For the moment, we will thus assume that selection is negligible, and the markers effectively neutral, and will provide further arguments supporting this assumption at the end of this section.

We used two methods to estimate N, both based in the change of genetic variance between the two sampling events.

The first method directly tracks changes in variance. The variance of the estimates of p and p′ between plants can be written as:
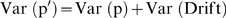
(2)The variance due to drift is equal to:
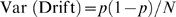
(3)Where *p* is the frequency of a given marker in initial populations. From Equations 2 and 3 we obtain:
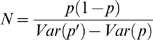
(4)The second technique is based on Fst statistics. Fst statistics were introduced by Wright [4] and represent a way to partition genetic variance within and between populations. One way to express Fst is:
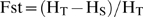
(5)where H_T_ is gene diversity assuming all populations form a single large population and H_S_ is the average gene diversity within each population. In our case, each plant represents a population. Gene diversities express the probability to randomly draw two different alleles and are thus equal to 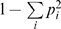, where *p_i_* is the frequency of each allele at the subdivision level under consideration. In our case H_T_ represents the gene diversity obtained after calculating the average frequency of each marker across all plants, while H_S_ represents the average across all plants of within plants gene diversities.

Using standard population genetics theory (e.g. [32]) it can be shown that, for a haploid such as a virus,
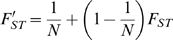
(6)where the prime denotes sampling at two different points in time. Rearranging Equation 6 yields an expression that can be used to estimate N:
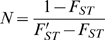
(7)We will refer to the estimates obtained by Equations 4 and 7 as N_V_ and N_F_ respectively.

To provide a confidence interval for these estimates we used a resampling technique. We bootstrapped over plants: for each bootstrap we randomly drew a sample of 50 plants with replacement, calculated Var(p′), Var(p) and p, and from that estimated N_V_. We also calculated H_T_ and H_S_, and from that estimated N_F_. We repeated this procedure 1,000 times and constructed a distribution of N_V_ and N_F_. The 95% confidence intervals correspond to the 2.5 and 97.5 percentiles of these distributions. In several bootstraps, the final variance was smaller than the initial variance, or the final F_ST_ was smaller than the initial F_ST_, yielding negative values of N_V_ or N_F_. In such cases, our estimation method does not work. Such cases would correspond to conditions leading to convergence of marker frequencies across plants, i.e. conditions where drift is negligible, and thus N can be regarded as infinite. We conservatively discarded such cases from the calculation of the upper limit of the confidence interval.

The average changes in frequency of the markers proved very small (see Table 1). Neutrality tests applied to VIT1, 3 and 4, demonstrated that only VIT1 and 3 might be slightly selected for and against, respectively (data not shown). Nevertheless, both these variants were used for estimating *N* because, while directional selection would lead to a situation where marker frequencies would be more similar across plants at the second sampling event than at the first, yielding smaller final variance and F_ST_, our data show the opposite, strongly contradicting the directional selection hypothesis. Another *a posteriori* justification is that while the neutrality tests indicate that one marker is slightly selected for, another is slightly selected against and another is effectively neutral, the N_V_ estimates obtained form all three are of the same order of magnitude. These arguments suggest that selection, if present, can be neglected relative to drift in our experiment.

**Table 1:** Genetic structure of initial and final CaMV populations.

	*Initial populations* a	*Final populations* a	E(Δp)^d^
	p^b^×10^2^ (SD^c^. 10^2^)	p′^b^×10^2^ (SD^c^. 10^2^)	
**VIT1**	41.6 (3.52)	45.8 (4.27)	4.2
**VIT2**	4.2 (1.90)	3.3 (1.55)	−0.9
**VIT3**	42.5 (4.19)	40.9 (5.08)	−1.6
**VIT4**	10.2 (2.88)	9.2 (3.19)	−1.0
**VIT5**	1.7 (1.89)	1.9 (1.61)	0.2
**VIT6**	1.7 (0.98)	3.6 (1.70)	1.9

The genetic structure of the 50 sampled CaMV populations is determined by the different markers present in each, as well as by their respective relative frequency. For reasons of clarity, only mean frequency values and standard deviations among the 50 repeats are shown here. The full dataset with details of the 50 CaMV populations is available (Table S1).

a Sampling protocol for initial and final populations is described in Materials and Methods.

b Mean relative frequency of each marker over the 50 replicates.

c Standard deviation of marker frequencies across the 50 replicates.

d Mean difference between initial and final frequencies in each replicate, and for each marker.

All values are expressed as percent of the viral genome population.

## Results

### Genetic structure of initial CaMV populations in whole plants

Following systemic spread of infection in plants inoculated with a mixture of 6 CaMV genetic variants (Mix6VIT), populations of viral genomes in each plant were purified from all fully expanded and systemically infected leaves, excluding inoculated leaves. DNA samples from each of 50 replicate plants were considered as the initial populations and were submitted to QSS analysis to detect markers in CaMV genomes and quantify their relative frequency as described in Materials and Methods. The mean frequency of each marker and the variance among the 50 CaMV populations collected at this stage is given in Table 1 (full dataset with details for each plant is provided in Table S1).

The six CaMV-VIT genotypes were found in widely unequal ratios in all 50 cases, with mean relative frequencies being close to 0 for CaMV-VIT5 and -VIT6, and up to around 40% for CaMV-VIT1 and -VIT3. However, a striking observation was that each marker was found with a very similar frequency in all 50 replicate CaMV populations (Table S1), as demonstrated by the very low standard deviation among repeats (Table 1). This result clearly indicates that little stochastic variation is generated by the inoculation process, or between the inoculation process and the sampling of initial populations. The frequency differences between markers being very consistent in all 50 replicates, they are certainly due to a non-stochastic phenomenon (discussed below).

### Genetic structure of final CaMV populations in single leaves

At late infection stages, 32 days after collecting the initial populations, a single systemically infected leaf per individual plant, randomly chosen between the 5^th^ and the 12^th^ new expanded leaves, was harvested. The CaMV populations extracted from these sampled leaves are considered as “final populations,” and their genetic structure (Table 1 and S1) was evaluated in exactly the same manner as that of the initial populations.

Remarkably, in all 50 repeats, the final population resembled the corresponding ancestor population despite the different sampling process (individual leaves for final populations versus pools of leaves for initial populations), demonstrating that very little change had occurred over this considerable time period, whatever the position or age of the analyzed leaf (see E(Δp) values [Table 1], and compare p and p′ values [Table S1] for each of the 50 plants). Consequently, as between initial populations, there were only small variations between the final populations, although the standard deviation was slightly higher in the latter case [Table 1].

Taken together, these results argue for the absence of large stochastic variations in the genetic structure of a CaMV population upon progression of the infection into newly formed leaves. This suggests that the effective size (*Ne*) of CaMV populations during host systemic colonization is likely to be surprisingly large (compared to data previously published on other plant viruses) as specifically evaluated in the next section.

### Estimation of bottleneck size in CaMV populations during systemic leaf colonization

Because the average relative frequency of markers CaMV-VIT2, -VIT5 and -VIT6 was close to, or even below, the limit of accuracy of the QSS method [30], their quantification was considered poorly reliable, and hence these markers were not used for further analysis. We thus used only the three markers -VIT1, -VIT3 and -VIT4 to estimate the average number of genomes founding the population in each leaf (N), as described in the Materials and Methods.

The estimates of N from these three markers yielded remarkably high values, corresponding to several hundreds of viral genomes. Two different statistical methods were applied to the data set and provided very consistent results, both for the observed bottleneck size and for the limit of the 95% confidence interval (compare N_V_ in Table 2, and N_F_ in Table 3). The lower limit of the confidence interval was >100 in all cases, whereas the higher limit reached thousands.

**Table 2:** Number of CaMV genomes founding the population in individual leaves, estimated through the analysis of the variance of the frequency of markers.

Marker^a^	Var(p′)×10^4^	Var(p)×10^4^	N_V_ b	95% confidence interval
**VIT1**	18.13	12.39	423.00	180–5034
**VIT3**	25.79	17.60	298.45	122–3219
**VIT4**	10.19	8.30	484.61	166–5193

a Only markers VIT1, VIT3, and VIT4 were used for estimating Nv, for reasons explained in the text.

b N_v_ (and 95% confidence interval) is calculated according to Equation 4 (see Materials and Methods), where p is estimated from values given in Table 1.

**Table 3:** Number of CaMV genomes founding the population in individual leaves, estimated through Fst statistics.

	*Initial populations* a	*Final populations* a
**H_T_**	0.63530089	0.61474821
**H_S_**	0.6314719	0.6093363
**Fst**	0.006027	0.008803
**N_F_ = 358 (184–1909)** b		

a Only markers VIT1, VIT3, and VIT4 were used for estimating N_F_, for reasons explained in the text.

b N_F_ was estimated from Fst values using Equation 7 (see Materials and Methods). The confidence interval, given in parentheses, was estimated by bootstrapping over plants.

## Discussion

This report evaluates the effective size of CaMV populations during systemic invasion of plant leaves. Several previous studies have used distinct experimental protocols to tackle similar questions with various viral species; the specifics and rationale of the protocol used here are discussed below. To eliminate variations in the genetic structure of viral populations that could be related to the inoculation process, the virus population present in the whole plant (excluding the inoculated leaves) soon after systemic spread of infection was considered as the starting point of the experiment, putative subsequent changes thus occurring only under the influence of within-plant processes. At this initial stage, the virus population uploaded into, and circulating within, the vascular system is most likely best represented by the overall content of the systemically infected leaves, as they have either received viruses from the vasculature, exported viruses into it, or both. Expanded infected leaves were therefore harvested for initial analysis, carefully preserving two to three young newly expanding leaves on the still-growing plant. The infected plants were then left to grow for a period of 32 days, during which the virus population successively colonized 10 to 15 emerging and expanding new leaves. Regardless of where the virus population originates from during this process (vascular system and roots at the beginning, then increasing numbers of leaves later on), analysis of single leaf (between positions 5 and 12 above the initial harvest) contents collected 32 days later should reveal the existence of any putative bottlenecks at any stage of the systemic infection. Indeed, final CaMV populations result largely from the successive leaf-to-leaf passages that occur sequentially when young sink leaves are infected, become sources and subsequently export virus into new sinks.

All genetic variants (CaMV-VIT1 to CaMV-VIT6) were co-inoculated at similar locations and at the same time point onto the two first true leaves of turnip plantlets. The reason why the relative proportions of the six variants are highly unequal later in systemically infected plants was mostly that unequal proportions were already present in the initial inoculum, Mix6VIT (further discussed in Table S2). Previous studies, based on co-infection of turnip plants by two distinct CaMV variants with seemingly equal growth rate, have compared the variant ratios in the initial inoculum and in resulting systemically infected plants. No differences were observed when concentrated virus particles were used for inoculation [33], whereas stochastic fluctuations were detectable when inoculum consisted in viral DNA prepared from infectious clones [34], presumably due to the lower infectivity of DNA preparations, engendering a stochastic founding effect in the latter case. These two studies indicate that indeed the inoculation process could induce unwanted fluctuations in repeated inoculations and prompted us to use virus particles enriched preparation for the purpose of our experiment (albeit development of systemic infection in between the two observation time points chosen in our protocol should not be affected by inoculation variations). Consistently, all 50 test plants in our study contained similar ratios of the six variants, suggesting very little stochastic variation during the inoculation process.

The values of p′ were estimated from individual leaf samples, each collected on a different test plant at a random position (between leaf position 5 and 12 above the initial harvest point). The remarkable observation that only relatively small variations in p′ are recorded in leaves from different plants (see standard deviation in Table 1) strongly indicates that very little variation occurs between leaves of the same plant. We therefore conclude that a similarly large founding population of several hundred viral genomes colonizes each newly formed leaf in plants systemically infected by CaMV.

The number of founder CaMV genomes in systemically infected leaves appears to be at least 100-fold higher than that determined in previously published studies for various RNA viruses [25]–[27]. Although there are clear differences in the inoculation and sampling protocols described in different studies, the presence of severe bottlenecks in WSMV [26], TMV [27], CMV [24] and PPV [25], and their obvious absence in CaMV, can hardly be explained by artifacts of experimental designs. Indeed, at least in the present study and in three of the cited examples [24]–[26], viruses moved not only from inoculated to systemic tissues, but also from systemically infected tissues to newly formed leaves or tiller. As mentioned in the Introduction, the previous repeated demonstration that different viruses undergo extreme bottlenecks during systemic infection of their host plant suggested that unavoidable barriers (e.g. connections between cells, etc.) may exist similarly for all plant viruses. The results presented here clearly demonstrate that such putative limiting barriers can be surmounted by some viruses and that the size of the viral population circulating *in planta* might thus be directly or indirectly controlled and needs to be evaluated for each virus individually. Whether the absence of severe bottlenecks demonstrated here for CaMV is common, and whether this phenomenon is related to the biology of the host plant, that of the virus, or more intricately on specific virus-host associations remains an open question.

The situation described here for CaMV re-opens the question as to what phenomena actually generate bottlenecks during virus infection. A reasonable hypothesis explaining the presence or absence of severe demographic bottlenecks would be the regulation of multiple infection of cells by several genomes of the viral population, in other words, regulation of the multiplicity of infection (MOI) of host cells. Indeed, it was proposed recently that bottlenecks seen in a PPV population infecting a *Prunus* tree could stem from the fact that viral genomes cannot secondarily invade tissues that have already been infected by a closely related genome [25], thus preventing extensive mixing of genetic variants within host. Consistently, two isogenic PPV genomes, but expressing fluorescent proteins of different colours, revealed their mutual exclusion within infected tissues, with apparently rare co-infection of single cells [35], a phenomenon also reported for other virus species, such as WSMW [26], TMV [36], and a few other RNA viruses [37]. This phenomenon could logically induce a very low MOI, in turn engendering bottlenecks (and small *Ne*), due to competition for host “territories” between variants of the same population. Accordingly, under this hypothesis, the absence of severe bottlenecks found for CaMV would suggest a higher MOI that is totally consistent with the remarkably high within-host recombination rate described for this virus [38], recombination being possible solely in multiply infected cells. We believe that explaining, at least in part, variations of within-plant bottlenecks in different virus species by their different capacity to multiply infect host cells is a very appealing hypothesis that will stimulate further novel research, the “natural” MOI during infection of a multi-cellular host being virtually unknown in viruses of both animals and plants.

## Supporting Information

Table S1 Whole dataset from the 50 replicate infected plants. p represents the marker relative frequency in initial populations. p′ represents the marker relative frequency in final populations. All values are indicated as percentage of the viral genome population. -, marker not detected.
(127 KB DOC)

Table S2 Analysis of the initial inoculum Mix6VIT. ^a^The mixture Mix6VIT was prepared and analysed by QSS as described in Materials and Methods. ^b^p_0_ = mean relative frequency determined from five independent repeats of QSS measurement. ^c^Standard deviation calculated from the five repeated QSS analysis. All values are expressed as percent of the viral genome population. The distribution of the relative frequencies of all VIT1-6 variants in the initial Mix6VIT inoculum was globally similar to that in the 50 inoculated plants (VIT1≈VIT3>VIT4>VIT2>VIT5≈VIT6). Nevertheless, apart from VIT1 and VIT3 which increased by about 10% in frequency from inoculum to infected plants, all other variants equally decreased by approximately 5% (compare p_0_ values in Table S2 and p values in Table 1). This phenomenon remains unclear and could be due to several different explanations, as for example: i) selection acting specifically at inoculation, could favour or disfavour some of the variants, ii) an undetermined threshold effect at the inoculation step could have positively and negatively affected the most and less frequent variants, respectively; iii) each variant in Mix6VIT originating from a different plant extract, they might have been differentially infectious due to unwanted and unequal damages of virus particles during extraction. Nevertheless, it is important to note that these considerations concern only the inoculation step, as only very minute changes in the mean frequency of all markers were detected later, over the 32 days separating the initial and final populations (see E(Δp) values in Table 1).
(32 KB DOC)
