# Supplementary material for: Large Bottleneck Size in Cauliflower Mosaic Virus Populations during Host Plant Colonization
Source: PLoS Pathog. 2008 Oct 10;4(10):e1000174. doi: 10.1371/journal.ppat.1000174 (PMC2553192; doi:10.1371/journal.ppat.1000174)
Supplement: Table S1 — Whole dataset from the 50 replicate infected plants. p represents the marker relative frequency in initial populations. p′ represents the marker relative frequency in final populations. All values are indicated as percentage of the viral genome population. -, marker not detected. (127 KB DOC) [file ppat.1000174.s001.doc]

**Table S1:** Whole dataset from the 50 replicate infected plants

|  | Plant N° | **1** | **2** | **3** | **4** | **5** | **6** | **7** | **8** | **9** | **10** | **11** | **12** | **13** | **14** | **15** | **16** | **17** | **18** | **19** | **20** | **21** | **22** | **23** | **24** | **25** |
| --- | --- | --- | --- | --- | --- | --- | --- | --- | --- | --- | --- | --- | --- | --- | --- | --- | --- | --- | --- | --- | --- | --- | --- | --- | --- | --- |
| **p** | VIT 1 | 46.1 | 38.8 | 45.7 | 44.7 | 43.2 | 41.1 | 48.7 | 43.2 | 41.6 | 44.9 | 42.9 | 45.9 | 44.6 | 37.5 | 40.5 | 41.8 | 41.7 | 35.1 | 39.2 | 45.4 | 41.5 | 43.5 | 38.9 | 40.5 | 39.0 |
|  | **VIT 2** | 4.7 | 3.7 | 0.7 | 3.3 | 5.0 | 3.9 | 4.3 | 8.6 | 2.3 | 4.6 | 3.0 | 5.3 | 4.5 | 3.9 | 3.8 | 4.1 | 3.8 | 5.4 | 2.1 | 3.2 | 4.0 | 3.2 | 12.1 | 2.2 | 4.9 |
|  | **VIT 3** | 39.1 | 44.6 | 46.8 | 37.1 | 37.7 | 39.9 | 40.4 | 34.8 | 47.4 | 39.6 | 42.6 | 42.3 | 42.5 | 48.7 | 44.7 | 40.9 | 44.2 | 50.4 | 49.7 | 42.2 | 44.1 | 44.7 | 33.7 | 44.1 | 42.3 |
|  | **VIT 4** | 10.2 | 10.4 | 6.7 | 13.2 | 11.6 | 11.9 | 5.3 | 12.2 | 8.5 | 9.4 | 10.2 | 6.2 | 8.1 | 8.6 | 9.4 | 11.5 | 10.3 | 9.1 | 9.1 | 9.2 | 8.9 | 8.6 | 8.1 | 13.2 | 12.5 |
|  | **VIT 5** | - | - | - | - | - | 2.7 | - | - | - | - | - | 0.3 | 0.3 | 1.3 | 0.6 | 0.2 | - | - | - | - | - | - | 7.1 | - | - |
|  | **VIT 6** | - | 2.5 | - | 1.7 | 2.4 | 0.5 | 1.3 | 1.2 | 0.4 | 1.5 | 1.3 | - | - | - | 1.1 | 1.6 | - | - | - | - | 1.4 | - | - | - | 1.3 |
|  |  |  |  |  |  |  |  |  |  |  |  |  |  |  |  |  |  |  |  |  |  |  |  |  |  |  |
| **p'** | **VIT 1** | 56.2 | 38.1 | 46.3 | 46.1 | 49.1 | 49.5 | 50.3 | 53.5 | 42.0 | 55.4 | 45.7 | 53.7 | 46.7 | 43.7 | 44.3 | 50.0 | 44.6 | 49.9 | 48.6 | 46.3 | 37.9 | 45.3 | 43.1 | 44.2 | 45.6 |
|  | **VIT 2** | - | 2.8 | 3.0 | - | 5.5 | 1.4 | 4.5 | - | - | - | 2.5 | 2.2 | 1.4 | 2.8 | 4.7 | 2.3 | 3.7 | 2.4 | 2.1 | 5.8 | 5.4 | 3.6 | 6.6 | - | - |
|  | **VIT 3** | 39.1 | 51.9 | 38.0 | 47.1 | 35.2 | 37.9 | 37.9 | 32.0 | 48.1 | 38.5 | 40.8 | 38.8 | 40.6 | 42.8 | 40.5 | 41.5 | 43.3 | 42.0 | 45.0 | 31.3 | 39.2 | 40.0 | 40.5 | 44.7 | 41.8 |
|  | **VIT 4** | 4.7 | 7.1 | 12.1 | 6.8 | 10.2 | 11.1 | 7.3 | 9.6 | 5.7 | 6.1 | 11.0 | 5.2 | 6.8 | 7.3 | 10.5 | 6.2 | 8.3 | 5.6 | 4.2 | 9.7 | 12.0 | 9.2 | 7.8 | 10.6 | 10.9 |
|  | **VIT 5** | - | - | - | - | - | - | - | 4.9 | 1.6 | - | - | - | 0.2 | - | - | - | - | - | - | 1.8 | 4.1 | 1.9 | 2.1 | 0.6 | 1.7 |
|  | **VIT 6** | - | - | 0.7 | - | - | - | - | - | 2.5 | - | - | - | 4.2 | 3.4 | - | - | - | - | - | 5.1 | 1.4 | - | - | - | - |
|  |  |  |  |  |  |  |  |  |  |  |  |  |  |  |  |  |  |  |  |  |  |  |  |  |  |  |
|  |  |  |  |  |  |  |  |  |  |  |  |  |  |  |  |  |  |  |  |  |  |  |  |  |  |  |
|  |  |  |  |  |  |  |  |  |  |  |  |  |  |  |  |  |  |  |  |  |  |  |  |  |  |  |
|  | Plant N° | **26** | **27** | **28** | **29** | **30** | **31** | **32** | **33** | **34** | **35** | **36** | **37** | **38** | **39** | **40** | **41** | **42** | **43** | **44** | **45** | **46** | **47** | **48** | **49** | **50** |
| **p** | VIT 1 | 42.8 | 37.6 | 31.9 | 40.1 | 37.9 | 39.5 | 40.5 | 44.0 | 42.7 | 45.4 | 43.8 | 43.9 | 43.7 | 41.1 | 37.3 | 40.0 | 36.4 | 47.6 | 41.1 | 43.5 | 42.1 | 42.0 | 48.2 | 37.9 | 34.2 |
|  | **VIT 2** | 1.3 | 3.0 | 6.2 | 4.5 | 4.8 | 0.4 | 3.1 | 3.2 | 6.8 | 5.5 | 3.6 | 2.6 | 4.9 | 3.9 | 6.4 | 6.3 | 4.2 | 4.1 | 3.7 | 6.1 | 2.8 | 2.7 | 4.9 | 3.7 | 6.0 |
|  | **VIT 3** | 50.0 | 37.4 | 39.1 | 45.0 | 43.4 | 54.3 | 44.6 | 40.8 | 40.1 | 40.1 | 43.4 | 45.0 | 41.0 | 42.9 | 45.8 | 43.4 | 43.6 | 35.7 | 40.1 | 39.8 | 48.2 | 43.2 | 35.2 | 41.5 | 39.0 |
|  | **VIT 4** | 5.8 | 20.4 | 20.8 | 9.7 | 11.3 | 5.8 | 9.5 | 11.3 | 10.4 | 8.9 | 9.1 | 8.5 | 9.3 | 10.5 | 10.1 | 10.0 | 11.3 | 9.9 | 12.3 | 10.5 | 6.9 | 10.2 | 9.5 | 14.4 | 12.6 |
|  | **VIT 5** | - | - | - | 0.5 | 2.6 | - | - | 0.7 | - | - | - | - | 1.1 | - | - | 0.3 | - | - | - | 0.2 | - | - | 2.1 | 2.5 | 4.9 |
|  | **VIT 6** | - | 1.6 | 2.0 | - | - | - | 2.4 | - | - | - | 0.1 | - | - | 1.6 | 0.3 | - | 4.5 | 2.6 | 2.8 | - | - | 1.9 | - | - | 3.3 |
|  |  |  |  |  |  |  |  |  |  |  |  |  |  |  |  |  |  |  |  |  |  |  |  |  |  |  |
| **p**' | **VIT 1** | 40.7 | 45.7 | 37.2 | 44.1 | 39.1 | 45.3 | 41.8 | 50.4 | 48.3 | 46.7 | 46.5 | 48.2 | 48.3 | 44.9 | 42.4 | 42.8 | 41.3 | 48.3 | 42.8 | 46.8 | 51.0 | 42.7 | 42.1 | 41.1 | 43.2 |
|  | **VIT 2** | - | 4.0 | 5.3 | 0.4 | 3.8 | - | 2.2 | 3.6 | 4.3 | 1.6 | 2.4 | 1.6 | 4.9 | 0.6 | 3.3 | 5.3 | - | 1.7 | 1.6 | 5.5 | 2.6 | 4.4 | 2.9 | - | - |
|  | **VIT 3** | 52.8 | 28.8 | 41.5 | 42.4 | 41.9 | 50.4 | 49.6 | 37.2 | 39.7 | 35.0 | 43.6 | 42.8 | 31.4 | 44.4 | 45.2 | 39.3 | 41.3 | 41.9 | 45.3 | 33.1 | 38.4 | 37.4 | 41.6 | 45.9 | 36.4 |
|  | **VIT 4** | 6.5 | 20.0 | 15.9 | 9.1 | 10.9 | 4.3 | 6.3 | 8.8 | 7.3 | 10.2 | 7.4 | 7.0 | 13.2 | 9.3 | 9.2 | 11.7 | 14.7 | 6.4 | 9.9 | 14.5 | 7.6 | 13.1 | 7.6 | 9.7 | 13.8 |
|  | **VIT 5** | - | 1.6 | 0.1 | - | 4.3 | - | - | - | 0.4 | 1.0 | - | 0.4 | 2.2 | 0.8 | - | 0.9 | 2.7 | 1.8 | 0.4 | - | 0.4 | 2.4 | - | 3.4 | 6.5 |
|  | **VIT 6** | - | - | - | 3.9 | - | - | - | - | - | 5.5 | - | - | - | - | - | - | - | - | - | - | - | - | 5.9 | - | - |

p represents the marker relative frequency in initial populations

p’ represents the marker relative frequency in final populations

All values are indicated as percentage of the viral genome population

- marker not detected
