# Supplementary material for: Large Bottleneck Size in Cauliflower Mosaic Virus Populations during Host Plant Colonization
Source: PLoS Pathog. 2008 Oct 10;4(10):e1000174. doi: 10.1371/journal.ppat.1000174 (PMC2553192; doi:10.1371/journal.ppat.1000174)
Supplement: Table S2 — Analysis of the initial inoculum Mix6VIT. aThe mixture Mix6VIT was prepared and analysed by QSS as described in Materials and Methods. bp0 = mean relative frequency determined from five independent repeats of QSS measurement. cStandard deviation calculated from the five repeated QSS analysis. All values are expressed as percent of the viral genome population. The distribution of the relative frequencies of all VIT1-6 variants in the initial Mix6VIT inoculum was globally similar to that in the 50 inoculated plants (VIT1≈VIT3>VIT4>VIT2>VIT5≈VIT6). Nevertheless, apart from VIT1 and VIT3 which increased by about 10% in frequency from inoculum to infected plants, all other variants equally decreased by approximately 5% (compare p0 values in Table S2 and p values in Table 1). This phenomenon remains unclear and could be due to several different explanations, as for example: i) selection acting specifically at inoculation, could favour or disfavour some of the variants, ii) an undetermined threshold effect at the inoculation step could have positively and negatively affected the most and less frequent variants, respectively; iii) each variant in Mix6VIT originating from a different plant extract, they might have been differentially infectious due to unwanted and unequal damages of virus particles during extraction. Nevertheless, it is important to note that these considerations concern only the inoculation step, as only very minute changes in the mean frequency of all markers were detected later, over the 32 days separating the initial and final populations (see E(Δp) values in Table 1). (32 KB DOC) [file ppat.1000174.s002.doc]

**Table S2:** Analysis of the initial inoculum Mix6VIT

|  | ***Relative Frequency****^a^* | ***Standart deviation*** |
| --- | --- | --- |
|  | p_0_^b^ x10^2^ | SD^c^. 10^2^ |
| VIT 1 | 28.1 | 1.5 |
| **VIT 2** | 10.1 | 3.7 |
| **VIT 3** | 30.1 | 2.3 |
| **VIT 4** | 16.7 | 0.9 |
| **VIT 5** | 7.0 | 1.4 |
| **VIT 6** | 7.7 | 0.7 |

^a^ The mixture Mix6VIT was prepared and analysed by QSS as described in the Materials and Methods.

^b^ p_0_ = mean relative frequency determined from five independent repeats of QSS measurement.

^c^ Standard deviation calculated from the five repeated QSS analysis.

All values are expressed as percent of the viral genome population.

The distribution of the relative frequencies of all VIT1-6 variants in the initial Mix6VIT inoculum was globally similar to that in the 50 inoculated plants (VIT1 ≈ VIT3 > VIT4 > VIT2 > VIT5 ≈ VIT6). Nevertheless, apart from VIT1 and VIT3 which increased by about 10% in frequency from inoculum to infected plants, all other variants equally decreased by approximately 5% (compare p_0_ values in table S2 and p values in Table 1). This phenomenon remains unclear and could be due to several different explanations, as for example: i) selection acting specifically at inoculation, could favour or disfavour some of the variants, ii) an undetermined threshold effect at the inoculation step could have positively and negatively affected the most and less frequent variants, respectively, iii) each variants in Mix6VIT originating from a different plant extract, they might have been differentially infectious due to unwanted and unequal damages of virus particles during extraction.

Nevertheless, it is important to note that these considerations concern only the inoculation step, as only very minute changes in the mean frequency of all markers were detected later, over the 32 days separating the initial an final populations (see E(∆p) values in Table 1).
